# Supplementary material for: Blocking Studies to Evaluate Receptor-Specific Radioligand Binding in the CAM Model by PET and MR Imaging
Source: Cancers (Basel). 2022 Aug 10;14(16):3870. doi: 10.3390/cancers14163870 (PMC9406147; doi:10.3390/cancers14163870)
Supplement: Supplementary file 1 [file cancers-14-03870-s001.zip › cancers-1853483-supplementary.pdf]

cancers

Article

Blocking Studies to Evaluate Receptor-Specific Radioligand Binding in the CAM Model by PET and MR Imaging

Jessica Löffler, Hendrik Herrmann, Ellen Scheidhauer, Mareike Wirth, Anne Wasserloos, Christoph Solbach, Gerhard Glatting, Ambros J. Beer, Volker Rasche and Gordon Winter

Supplements

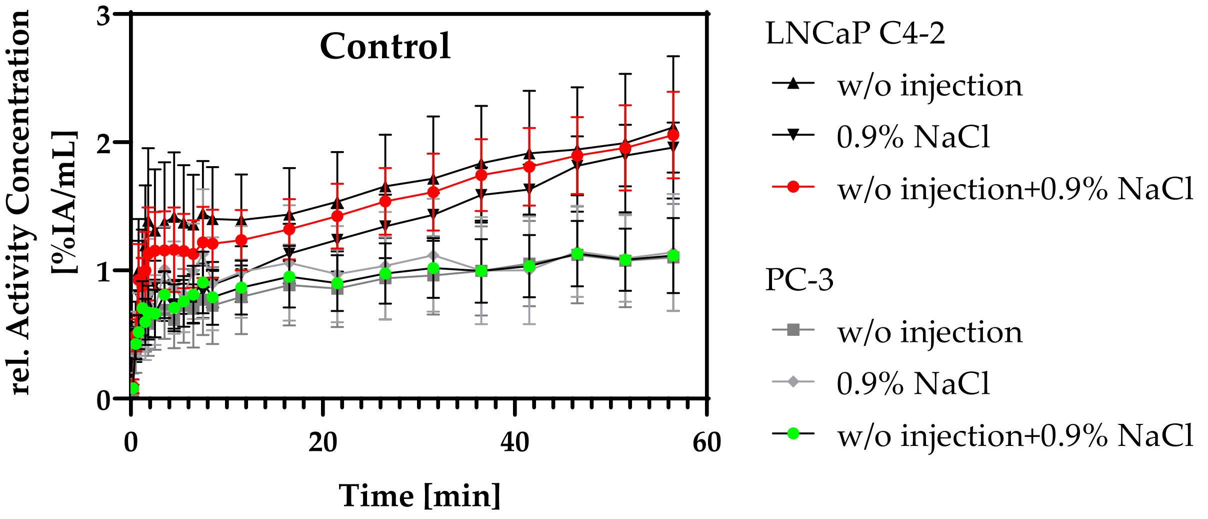

Figure S1. Comparison and summary of TACs of controls, with and without additional injection of 0.9% NaCl for the PSMA+ and the PSMA- tumor xenografts. While there are still substantial differences in the first 10 min post-injection, presumably due to perfusion, the curves become more and more similar during the measurement period. The increase and final accumulation do not show significant injection-dependent differences, which is why the data have been combined for further evaluation (red and green curve).

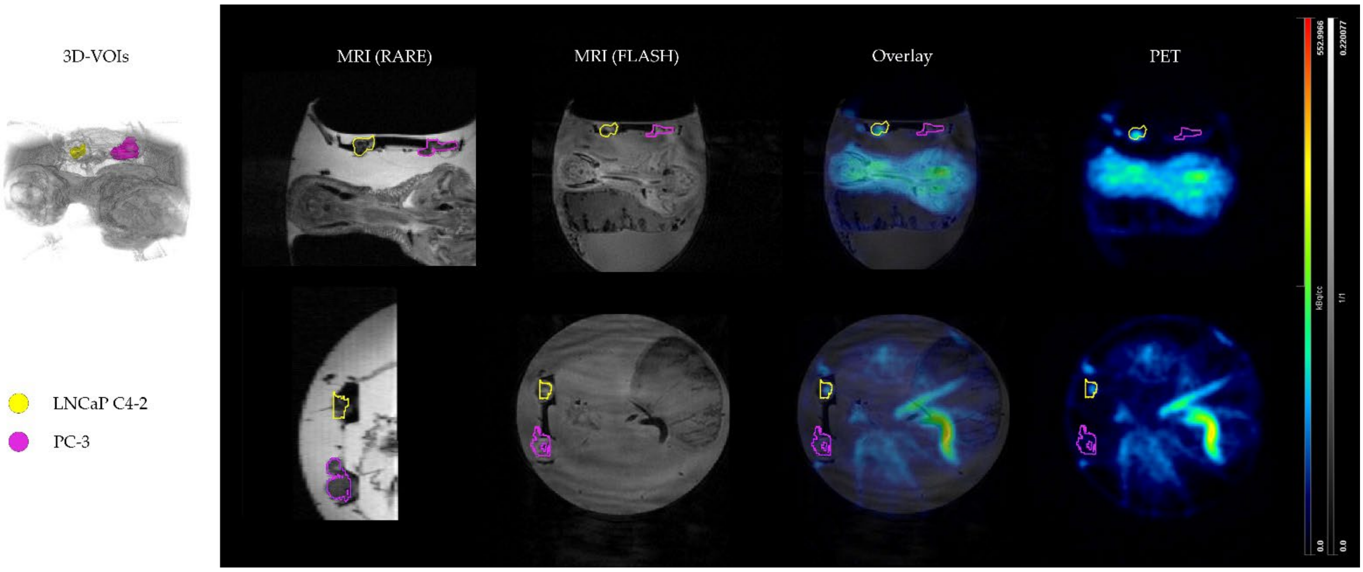

Figure S2. Example images for tumor VOI positioning with PMOD. VOIs were drawn based on the MRI (RARE) scans. MRI (RARE), MRI (Flash), and PET data were separately superimposed, so VOIs from RARE could also be used for the other scans. The original PMOD VOIs were mapped in the respective image sections to demonstrate reliable localization of the contours. In addition, a 3D image of the MRI scan (RARE), including the VOI regions, was added to illustrate the three-dimensional aspect of the selection.

cancers

MDPI

Table S1. Decay corrected TAC data [%IA/mL] based on PMOD evaluation.

	Control (no 2-PMPA) [%IA/mL]															
	Ctrl. 1		Ctrl. 2		Ctrl. 3		Ctrl. 4		Ctrl. 5		Ctrl. 6		Ctrl. 7		Ctrl. 8	
**Time**	**C4-2**	**PC-3**	**C4-2**	**PC-3**	**C4-2**	**PC-3**	**C4-2**	**PC-3**	**C4-2**	**PC-3**	**C4-2**	**PC-3**	**C4-2**	**PC-3**	**C4-2**	**PC-3**
10	0.064	0.027	0.441	0.187	0.041	0.059	0.022	0.018	0.025	0.035	0.154	0.201	0.007	0.056	0.021	0.069
30	0.876	0.311	0.871	1.037	0.348	0.390	0.089	0.076	0.207	0.071	0.513	0.772	0.936	0.563	0.118	0.181
50	2.061	0.574	1.810	1.094	0.814	0.483	0.158	0.202	0.152	0.068	0.386	1.035	1.656	0.509	0.376	0.193
70	1.692	0.341	1.918	1.997	0.624	0.889	0.429	0.273	0.188	0.388	0.622	0.981	1.068	0.556	0.474	0.224
90	0.831	0.495	2.063	1.664	0.338	0.582	2.537	0.200	0.217	0.223	0.584	0.919	1.022	0.620	0.392	0.090
110	0.890	0.746	1.911	1.499	0.484	0.218	3.354	0.158	0.308	0.297	0.541	1.655	0.937	0.550	0.610	0.251
150	0.837	0.505	1.726	1.698	0.477	0.528	3.005	0.201	0.516	0.308	0.813	1.202	1.243	0.615	0.609	0.261
210	1.124	0.566	1.619	1.573	0.437	0.419	3.063	0.293	0.623	0.599	0.583	1.525	1.177	1.114	0.631	0.396
270	1.190	0.431	1.819	1.494	0.377	0.348	3.158	0.420	0.546	0.378	0.435	1.433	1.086	0.970	0.686	0.193
330	0.855	0.554	1.964	1.854	0.512	0.449	2.849	0.494	0.685	0.266	0.684	1.298	1.012	0.877	0.641	0.271
390	1.149	0.604	2.007	1.882	0.563	0.349	2.506	0.496	0.574	0.170	0.492	1.512	1.010	1.206	0.720	0.267
450	1.003	0.497	2.061	1.842	0.593	0.507	2.737	0.715	0.839	0.295	0.566	1.544	1.258	1.717	0.698	0.125
510	1.084	0.428	2.158	1.895	0.523	0.429	2.556	0.648	0.680	0.231	0.815	1.451	1.228	1.023	0.623	0.216
690	1.103	0.717	2.126	1.911	0.625	0.526	2.346	0.571	0.767	0.247	0.877	1.543	1.387	1.104	0.650	0.320
990	1.031	0.826	2.457	2.092	0.641	0.597	2.144	0.648	0.907	0.264	1.183	1.840	1.503	1.053	0.707	0.282
1290	1.029	0.789	2.853	2.020	0.687	0.484	1.947	0.646	1.165	0.355	1.214	1.607	1.680	0.998	0.820	0.309
1590	1.320	0.883	3.091	2.149	0.814	0.614	1.945	0.786	1.117	0.265	1.449	1.668	1.711	1.197	0.868	0.242
1890	1.194	0.832	3.526	2.134	0.797	0.646	1.913	0.823	1.156	0.367	1.470	1.758	1.714	1.324	1.121	0.273
2190	1.536	0.881	3.532	2.338	0.902	0.585	1.815	0.834	1.399	0.340	1.613	1.696	1.953	1.048	1.203	0.254
2490	1.592	0.986	3.751	2.325	0.900	0.714	1.906	0.869	1.423	0.377	1.845	1.745	1.805	0.963	1.244	0.294
2790	1.568	0.958	3.762	2.586	0.955	0.719	1.997	0.922	1.435	0.425	1.961	1.782	2.123	1.110	1.363	0.551
3090	1.360	0.913	4.082	2.490	1.051	0.654	1.920	0.866	1.553	0.462	1.974	1.677	2.266	1.093	1.446	0.511
3390	1.751	0.943	4.235	2.719	0.997	0.711	2.029	0.828	1.570	0.309	2.236	1.865	2.058	1.261	1.583	0.294

Table S2. Decay corrected TAC data [%IA/mL] based on PMOD evaluation.

0.005 µM 2-PMPA [%IA/mL]										
	1		2		3		4		5	
**Time**	**C4-2**	**PC-3**	**C4-2**	**PC-3**	**C4-2**	**PC-3**	**C4-2**	**PC-3**	**C4-2**	**PC-3**
10	0.062	0.015	0.029	0.022	0.029	0.024	0.080	0.095	0.038	0.096
30	0.246	0.087	0.785	1.893	0.300	0.190	0.604	0.497	0.326	0.198
50	1.725	0.417	0.915	1.459	0.770	0.034	0.265	0.397	0.999	0.532
70	1.054	0.462	0.820	1.026	0.793	0.393	0.421	0.112	0.611	0.475
90	1.185	0.181	0.311	0.594	0.936	0.141	0.198	0.144	0.777	0.606
110	0.805	0.364	0.885	0.736	1.087	0.360	0.455	0.082	0.884	0.268
150	0.952	0.288	0.796	1.056	1.060	0.176	0.382	0.193	0.559	0.430
210	1.278	0.356	0.706	1.011	1.092	0.117	0.496	0.157	0.535	0.255
270	0.940	0.304	0.985	0.984	0.997	0.334	0.270	0.061	0.703	0.368
330	1.067	0.379	1.045	0.940	1.196	0.545	0.374	0.343	0.571	0.322
390	1.141	0.360	1.090	0.780	1.670	0.439	0.516	0.129	0.869	0.313
450	1.458	0.237	1.034	1.152	1.630	0.171	0.439	0.080	0.649	0.451
510	1.443	0.375	1.342	1.151	1.939	0.508	0.324	0.087	0.767	0.491
690	1.487	0.480	1.444	1.059	1.835	0.325	0.324	0.238	1.141	0.497
990	1.923	0.623	1.561	1.278	1.652	0.253	0.422	0.189	1.220	0.456
1290	2.100	0.785	1.848	1.261	1.487	0.286	0.609	0.205	1.645	0.615
1590	2.144	0.813	1.972	1.295	1.386	0.301	0.426	0.121	1.698	0.688
1890	2.353	0.732	2.045	1.455	1.401	0.464	0.498	0.121	1.895	0.665
2190	2.404	0.977	2.176	1.455	1.590	0.326	0.442	0.215	1.870	0.661
2490	2.560	1.051	2.317	1.530	1.594	0.476	0.488	0.237	1.791	0.671
2790	2.237	0.966	2.471	1.519	1.427	0.503	0.501	0.204	2.129	0.832
3090	2.378	0.967	2.550	1.406	1.395	0.515	0.536	0.187	2.216	0.677
3390	2.418	1.065	2.675	1.391	1.542	0.473	0.730	0.364	1.857	0.627

Table S3. Decay corrected TAC data [%IA/mL] based on PMOD evaluation.

0.05 μM 2-PMPA [%IA/mL]										
	1		2		3		4		5	
**Time**	**C4-2**	**PC-3**	**C4-2**	**PC-3**	**C4-2**	**PC-3**	**C4-2**	**PC-3**	**C4-2**	**PC-3**
10	0.026	0.037	0.173	0.036	0.024	0.175	0.328	0.031	0.023	0.401
30	0.267	0.703	1.650	0.162	0.152	0.445	0.442	0.267	0.349	1.056
50	3.154	2.034	1.474	0.667	0.726	0.587	1.132	1.645	0.979	0.907
70	2.803	2.322	1.680	0.777	0.600	0.327	1.784	1.739	0.731	0.922
90	2.700	1.474	0.862	0.892	0.727	0.200	2.156	1.639	0.558	1.286
110	2.754	1.492	0.719	0.368	0.419	0.523	1.785	0.945	0.783	0.698
150	2.629	1.683	0.728	0.835	0.598	0.401	2.034	1.585	1.048	0.980
210	2.315	1.971	0.963	0.659	0.846	0.809	1.913	1.327	0.752	0.947
270	2.463	1.885	0.790	0.487	0.481	0.655	1.972	1.732	0.998	0.835
330	2.910	2.012	0.675	0.759	0.534	0.570	1.905	1.385	1.038	0.851
390	2.513	1.796	0.789	0.577	0.308	0.386	1.928	1.287	1.011	0.948
450	2.613	2.181	0.792	0.374	0.681	0.650	1.838	1.397	0.986	0.809
510	2.389	2.179	0.677	0.493	0.860	0.456	1.869	1.344	0.837	0.766
690	2.849	2.103	0.889	0.445	0.824	0.848	2.060	1.506	1.378	1.031
990	2.764	2.337	0.900	0.361	0.712	0.722	2.273	1.747	1.728	1.075
1290	2.562	2.625	1.227	0.376	0.949	0.936	2.584	1.913	1.922	1.002
1590	2.669	2.703	1.229	0.350	0.817	1.317	2.707	1.991	1.932	1.103
1890	2.766	2.876	1.591	0.371	0.858	1.432	2.863	2.256	2.424	1.404
2190	2.782	3.022	1.679	0.465	1.036	1.464	2.998	2.250	2.873	1.444
2490	3.007	3.185	1.369	0.596	1.132	1.612	3.288	2.184	3.318	1.445
2790	3.222	2.751	1.626	0.469	1.362	1.599	3.293	2.660	3.382	1.460
3090	3.389	2.779	1.554	0.627	1.377	1.466	3.366	2.523	3.515	1.389
3390	3.377	2.890	1.515	0.631	1.312	1.477	3.479	2.509	3.192	1.348

Table S4. Decay corrected TAC data [%IA/mL] based on PMOD evaluation.

0.5 μM 2-PMPA [%IA/mL]								
	**1**		**2**		**3**		**4**	
**Time**	**C4-2**	**PC-3**	**C4-2**	**PC-3**	**C4-2**	**PC-3**	**C4-2**	**PC-3**
10	0.202	0.627	0.020	0.009	0.158	0.012	0.072	0.000
30	0.697	1.352	1.987	0.270	0.637	0.469	0.617	1.873
50	0.490	1.830	2.810	0.842	1.086	1.174	1.136	2.240
70	0.636	1.474	1.357	1.181	1.821	0.954	0.895	1.372
90	1.068	0.639	1.685	0.846	1.305	0.645	0.990	1.314
110	0.376	1.023	1.838	0.785	1.240	0.901	0.849	2.095
150	0.757	0.975	1.535	0.768	1.075	1.049	0.955	1.098
210	0.736	0.860	1.462	0.785	0.999	1.334	0.624	1.064
270	0.727	1.000	1.228	0.735	0.582	0.986	1.042	0.418
330	0.877	0.848	1.137	0.949	0.616	1.108	0.655	1.244
390	0.924	1.067	1.353	1.050	0.447	1.187	0.790	0.971
450	0.845	0.772	1.154	0.987	0.718	0.509	0.745	0.675
510	0.847	0.744	1.092	1.224	0.407	0.727	1.039	1.169
690	1.132	1.194	1.260	1.112	0.581	0.642	0.841	1.175
990	1.447	1.275	1.073	1.273	0.652	0.720	1.062	1.258
1290	1.433	1.358	0.953	1.629	0.732	0.899	1.358	1.363
1590	1.360	1.402	0.913	1.784	0.845	0.806	1.319	1.189
1890	1.426	1.544	0.900	1.443	0.767	0.390	1.372	1.329
2190	1.537	1.718	0.830	1.678	0.914	0.756	1.442	1.477
2490	1.641	1.798	0.894	1.613	0.821	0.751	1.444	1.495
2790	1.566	1.646	0.892	1.653	0.910	0.914	1.455	1.706
3090	1.970	1.827	1.062	1.825	1.024	0.619	1.679	1.581
3390	1.699	1.857	0.994	1.611	0.822	0.771	1.405	1.471

Table S5. Decay corrected TAC data [%IA/mL] based on PMOD evaluation.

50 μM 2-PMPA [%IA/mL]										
	1		2		3		4		5	
**Time**	**C4-2**	**PC-3**	**C4-2**	**PC-3**	**C4-2**	**PC-3**	**C4-2**	**PC-3**	**C4-2**	**PC-3**
10	0.080	0.050	0.012	0.011	0.079	0.015	0.025	0.046	0.035	0.017
30	0.026	0.074	0.039	0.055	0.276	0.073	0.073	0.141	1.127	0.492
50	0.215	0.182	0.333	0.925	9.388	0.138	0.046	0.117	1.692	1.269
70	0.778	1.572	0.727	0.987	4.062	0.802	0.269	0.131	2.283	1.489
90	1.004	1.278	1.015	0.893	3.916	1.230	0.177	0.265	2.185	1.095
110	0.545	1.149	0.766	0.952	2.491	0.837	0.122	0.140	1.664	1.374
150	0.890	1.340	0.820	0.663	2.730	0.604	0.163	0.194	1.677	1.310
210	0.742	0.831	0.557	0.710	2.758	0.498	0.147	0.144	1.625	1.347
270	0.839	0.934	0.777	0.826	2.744	0.526	0.131	0.162	1.870	1.157
330	1.159	1.206	0.621	1.024	2.374	0.508	0.153	0.139	1.534	1.321
390	0.650	1.210	0.894	1.152	2.315	0.670	0.151	0.243	1.159	1.200
450	0.685	0.949	0.711	0.752	2.583	0.512	0.224	0.153	1.345	1.294
510	1.179	1.059	0.812	0.896	2.283	0.676	0.185	0.225	1.351	1.262
690	0.947	1.276	0.818	0.782	2.793	0.670	0.186	0.207	1.512	1.359
990	1.301	1.229	0.722	0.876	2.767	0.740	0.201	0.259	1.588	1.572
1290	1.238	1.555	0.729	0.759	2.514	0.804	0.324	0.279	1.684	1.576
1590	1.260	1.343	0.598	0.979	2.703	0.934	0.334	0.342	1.974	1.696
1890	1.545	1.617	0.874	1.013	2.407	1.023	0.392	0.321	2.093	1.759
2190	1.853	1.436	0.914	0.926	2.267	1.339	0.415	0.424	2.069	1.930
2490	1.980	1.635	1.047	1.039	2.123	1.486	0.370	0.350	2.115	2.208
2790	2.241	1.300	1.025	1.025	2.272	1.707	0.391	0.397	2.090	2.156
3090	2.504	1.597	1.291	1.100	2.469	1.668	0.505	0.333	2.297	2.227
3390	2.250	1.683	1.057	1.214	2.392	2.014	0.414	0.356	2.295	2.025
